# The Effect of Sea Surface Slicks on the Doppler Spectrum Width of a Backscattered Microwave Signal

**DOI:** 10.3390/s8063780

**Published:** 2008-06-06

**Authors:** Vladimir Karaev, Mikhail Kanevsky, Eugeny Meshkov

**Affiliations:** Institute of Applied Physics, Russian Academy of Science, 46 Ulyanov Str., Nizhny Novgorod, 603950, Russia

**Keywords:** remote sensing, ocean surface, slick, microwave radar, Doppler spectrum

## Abstract

The influence of a surface-active substance (SAS) film on the Doppler spectrum width at small incidence angles is theoretically investigated for the first time for microwave radars with narrow-beam and knife-beam antenna patterns. It is shown that the requirements specified for the antenna system depend on the radar motion velocity. A narrow-beam antenna pattern should be used to detect slicks by an immobile radar, whereas radar with a knife-beam antenna pattern is needed for diagnostics from a moving platform. The study has revealed that the slick contrast in the Doppler spectrum width increases as the radar wavelength diminishes, thus it is preferable to utilize wavelengths not larger than 2 cm for solving diagnostic problems. The contrast in the Doppler spectrum width is generally weaker than that in the radar backscattering cross section; however, spatial and temporal fluctuations of the Doppler spectrum width are much weaker than those of the reflected signal power. This enables one to consider the Doppler spectrum as a promising indicator of slicks on water surface.

## 1. Introduction

The detection and recognition of sea surface slicks by means of remote facilities grow more and more urgent because of aggravating environmental situation and increasing sea surface pollution by human activity products. An important advantage of radar methods compared to optical ones is their independence of weather and light conditions.

Both radars and radiometers are employed to diagnose surface-active substance (SAS) films on the sea surface.

Modern methods using data of active microwave radars (synthetic aperture radars, side-looking radars, and scatterometers) are based on the effect of a spectral density decrease of small scattering ripples in a slick [1–5]. In the first approximation, large-scale waves are not sensitive to the presence of slicks on the sea surface.

In the range of middle incidence angles one deals with the Bragg scattering mechanism and the power of the reflected radar signal is proportional to the spectral density of resonance ripples, i.e., as ripples enhance, the reflected signal power grows, while as the ripple intensity decreases, the power reduces.

The contribution of large-scale roughness to the formation of the reflected radar signal consists in variation of the reflected signal power produced by slope and hydrodynamic modulation; besides, namely the statistical characteristics of large-scale waves determine the Doppler spectrum width.

A SAS film occurring on water surface leads to ripple damping (slick formation) and hence the reflected radar signal power (the radar backscattering cross section) reduces. Radar contrasts (clean water/slick) in the radar backscattering cross section can achieve tens dB.

Under the conditions of temporal instability (or spatial inhomogeneity) of wind speed, one observes significant fluctuations of the radar backscattering cross section caused by variance of the spectral density of small resonance ripples and related to the temporal or spatial variance of the near-surface wind speed. Small ripples rapidly respond to the wind speed variation and the spectral density of gravity-capillary waves is proportional to the wind speed. As a result “wind” slicks appear, i.e., parts with lower or higher spectral densities of small-scale waves than those in neighboring parts. Besides, spatial inhomogeneity of the spectral density of ripples is brought about by large-scale waves on the sea surface.

**Figure 1.**
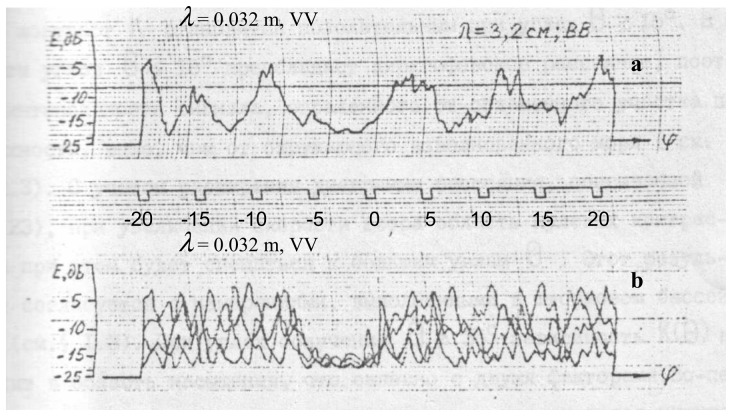
Record of signals scattered by the sea surface with oil products at moderate waves: a – one scan, b – four records obtained during two minutes. Grazing angle is approximately 1°-2° and azimuthal angles change from -20° to 20°.

Figure 1 shows the experimental results on slick detection (“smoothed” ripple part) under the conditions of moderate sea [6].

The authors employed the following technique. Oil products were poured out on the sea surface; the antenna system and the strobe were aimed at this place. Then sector scanning was realized by the antenna system, so that the oil film was at the center of the viewing area. Azimuth marks *φ* were recorded at intervals of 5 degrees. The radar wavelength equaled 3.2 cm; the polarization was vertical.

It is seen from the figure that the average value of the scattered signal decreases at the location of the oil slick. Other deep amplitude modulations in fig. 1a are also seen in the records, which are caused by the reflected signal modulation due to large-scale waves.

A real slick can be revealed among these modulations, since the film position does not change; so the actual position of the slick can be determined by averaging over several scans (see fig. 1b). Unfortunately, repeated measurements are not always possible in practice.

Therefore, the above-mentioned effects impede the detection of slicks produced by a SAS film on the surface using the reflected signal power in the region of middle and large incidence angles.

The width and the shift of the Doppler spectrum of the radar backscattered signal are not obviously dependent on the resonance ripple intensity, which makes it difficult to develop methods of slick detection by spectral characteristics of the reflected signal. Any examples of applying such methods to the surface film diagnostics at the incidence angles close to nadir are unknown to us.

Our previous investigations devoted to the development of the algorithms of roughness parameter retrieval employing a microwave Doppler radar at small incidence angles [7–10] stimulated the study of the possibility of detecting films on water surface by the Doppler spectrum width.

The present paper deals with the influence of slicks on the Doppler spectrum width for the Bragg and quasi-specular backscattering mechanisms. The conditions of slick detection are discussed for resonance scattering. The dependence of the Doppler spectrum width on the measurement conditions at small incidence angles is analyzed. Numerical estimates of the influence of sea surface slicks on the Doppler spectrum width at small incidence angles for radars with narrow-beam (1° × 1°) and knife-beam (1° × 22°) antenna patterns are presented.

### 2. The SAS film influence on the wave spectrum. Results and discussion

To describe the SAS film influence on the surface wave spectrum, in the calculations we used the model suggested in [11].

SAS films on water surface efficiently damp short wind waves, i.e., slicks occur. The decrease of the spectral density depends on the film thickness and the properties of the substance.

To describe the variation of the spectral density of surface waves in the slick, we introduce a concept of contrast *K_slick_* as follows:

(1) $$K_{\text{slick}}=W\;(\vec{\kappa })/W_{0}\;(\vec{\kappa }),$$

where *W*_0_(*κ⃗*) is the wave spectrum on clean water, *W*(*κ⃗*) is the wave spectrum in the slick, and *κ* is the wavenumber.

Depending on the relation between the viscous damping decrement *χ* and the wind increment *β*, the contrast is calculated as [12]:

(2) $$\begin{array}{cc} K_{\text{slick}}=\frac{\beta -2\chi }{\beta -2\chi_{0}}, & \beta > \end{array}2\chi_{0}\text{and}\;\beta \;>2\chi$$

or

(3) $$\begin{array}{cc} K_{\text{slick}}=\frac{2\chi_{0}-\beta }{2\chi -\beta }, & \beta < \end{array}2\chi_{0}\text{and}\;\beta <2\chi .$$

If the above-mentioned conditions are not fulfilled, strong smoothening (damping) at this wavelength is observed; in this case *K_slick_* ≈ 0.

The expressions for the viscous damping decrement and the wind increment are given below [13-15]:

(4) $$\beta =(0.04\pm 0.02)\cdot \kappa^{2}\cdot u_{*}^{2}/\omega ,$$

and

(5) $$\chi =2\nu \kappa^{2}\frac{\frac{2\nu \kappa^{2}}{\omega }-\frac{E\kappa^{3}}{\rho \omega^{2}}\sqrt{\frac{2\nu \kappa^{2}}{\omega }}+\frac{E^{2}\kappa^{6}}{2\rho^{2}\omega^{4}}\sqrt{\frac{\omega }{2\nu \kappa^{2}}}}{\frac{2\nu \kappa^{2}}{\omega }-\frac{2E\kappa^{3}}{\rho \omega^{2}}\sqrt{\frac{2\nu \kappa^{2}}{\omega }}+\frac{2E^{2}\kappa^{6}}{\rho^{2}\omega^{4}}},$$

where u_*_ is the friction velocity (cm/s), $\omega =\sqrt{g\kappa +\sigma \kappa^{3}/\rho }$ is the dispersion relation for surface waves, *g* is the acceleration of gravity (cm/s^2^), *σ* is the surface tension coefficient (g/s^2^), *ρ* is the water density (g/cm), *ν* is the kinematic viscosity (cm^2^/s), and *E* is the film elasticity modulus (mN/m) dependent on the concentration and type of the substance, *χ*_0_ corresponds to the case of clean water.

The model described above was frequently employed to carry out numerical calculations, e.g., [16, 17] and its adequacy was validated.

In further numerical estimates we choose vegetable oil as SAS. This choice is explained by the necessity to provide environmental safety of the experiment, thus it is more preferable to use a substance that will not pollute the environment; and then the calculation results will be compared to the future experimental ones.

For the numerical estimates we utilize the following values: ***ν*** = 0.01 cm^2^/s, *σ* (water) = *72* g/s^2^, and *σ*(vegetable oil) = 40 g/s^2^.

The film elasticity *E* is in the range 5 ÷ 30 mN/m; in the calculation we use two values: 10 and 20 mN/m.

For convenience of comparison to the data obtained by other authors, the wind speed is assigned at a height of 10 m above the sea surface (*U*_10_); when calculating the friction velocity u_*_ for neutral stratification, the logarithmic profile of the wind speed is used [18]:

(6) $$U_{10}=\frac{u_{*}}{0.4}\ln(10/z_{0}),$$

where *Z*_0_ is the surface roughness parameter (height).

As the wave spectrum (elevation spectrum) we employ the model suggested by us in [19] and successfully used in numerical calculations. The formulas for the wind wave spectrum taking into account the case of developing wind waves are presented in Annex 1. This model is in good agreement with experimental data, for example, the Cox and Munk formula for slopes.

To pass over from the elevation spectrum to the slope spectrum, we invoke the known transformation implying multiplication of the elevation spectrum by the coefficient ***κ***^2^[20, 21].

**Figure 2.**
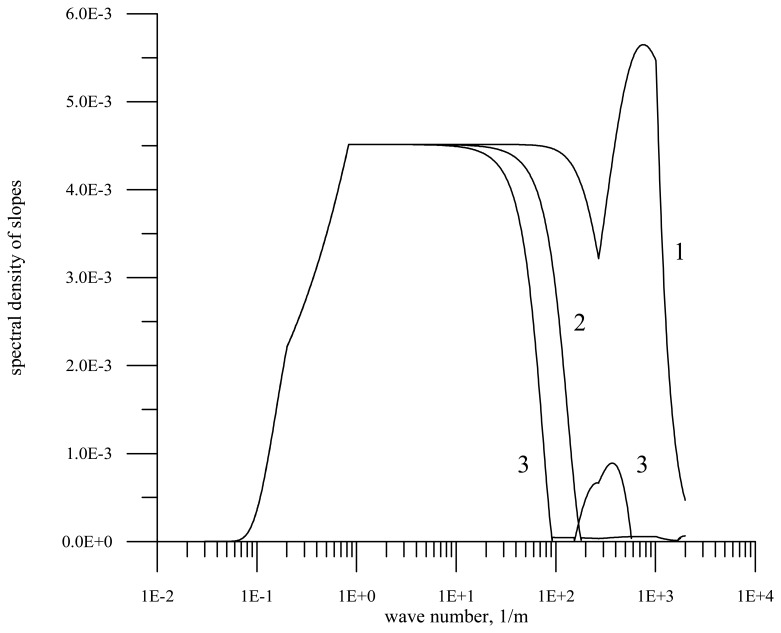
Spectra of slopes for the wind velocity 7 m/s. Curve 1 – the initial spectrum on clean water, curve 2 – the spectrum in the slick for the film elasticity *E* = 10 mN/m, curve 3 – the spectrum for the film elasticity *E* = 20 mN/m.

An example of the slope spectrum transformation in the slick for the wind speed 7 m/s is shown in fig. 2. The transformations of spectrum are calculated according to formulas (1) – (5).

The figure shows the slope spectrum on clean water and the spectra in the slick for the film elasticity *E* = 10 mN/m and 20 mN/m. The shown violation of monotonic decrease in the spectral density with the increasing wavenumber for the film viscosity 20 mN/m entirely corresponds to the known experimental data on the SAS influence on the surface wave spectrum (formulas (1) – (5)).

It is seen from the figure that the film damps short waves and thus statistical characteristics of waves vary. Since the main contribution to slope variance is made by short waves, the variation of the slope variance in the slick is strongest, while the variation of the wave height determined by large-scale waves free of the film influence is weakest.

## 3. Statistical characteristics of surface waves as applied to the problem of microwave backscattering at small incidence angles

To describe backscattering of centimeter electromagnetic waves at small incidence angles, the Kirchhoff method is used now [22, 23]. In this method it is assumed that scattering takes place in the parts of the large-scale wave profile oriented perpendicularly to the incident radiation. Small ripples on a large-scale wave induces diffusion scattering and reduces the backscattering power compared to the smooth surface.

The radar backscattering cross section along the *X*-axis for radar with a narrow-beam antenna pattern is calculated by the well-known formula:

(7) $$\sigma_{0}=\frac{{\left|R_{\text{eff}}\right|}^{2}}{2\sigma_{xx}\sigma_{yy}}\cdot \exp\left[-\frac{\tan^{2}\theta_{0}}{2\sigma_{xx}^{2}}\right]\;,$$

where $\sigma_{xx}^{2}$and $\sigma_{yy}^{2}$are slope variances along and across the wind direction, *θ*_0_ is the incidence angle, and *R_eff_* is the effective reflection coefficient introduced instead of the Fresnel coefficient to take into account the signal attenuation by small surface ripples [22-26].

Small ripples are suppressed in a slick, which leads to an increase of the effective reflection coefficient; besides, the slope variance decreases, which also results in a growth of the radar backscattering cross section.

The experimental data confirm the statement that at nadir probing the backscattering radar cross section in the slick can be by 2–4 dB higher than that on clean water [6, 27]. At middle incidence angles the inverse effect is observed: the radar backscattering cross section in the slick is smaller than that on clean water.

To pass over to numerical estimates of the slick influence on the statistical characteristics of large-scale waves, we shall divide arbitrarily the full ocean spectrum into small-scale and large-scale parts and introduce a boundary wavenumber ***κ****_b_*. The small-scale part ***κ****_b_* > ***κ*** is held obeying the perturbation theory, while the large-scale part requires conformity with physical optics [28]:

$$k{\text{Rcos}}^{3}\theta_{0}\gg 1,$$

R being the mean curvature radius, and *k* is the radar wavenumber. The latter inequality means that for electromagnetic diffraction the appropriate large-scale surface can be replaced at its arbitrary point by a tangent plane with a local normal.

The slope variance of large-scale waves is found by integration over the slope spectrum to the boundary wavenumber, i.e., the contribution is made by the waves located on the wavenumber axis to the left of the boundary wavenumber ***κ****_b_*.

Let us give formulas for the dependence of the boundary wavenumber on the wind speed for the two radar wavelengths (0.021 m and 0.008 m) calculated for our model of the wave spectrum:

$\kappa_{b}(\lambda =0.021)=171.844-29.9715\cdot U_{10}+11.0462\cdot U_{10}\ln(U_{10})--0.8727\cdot U_{10}^{2}+0.13932\cdot U_{10}^{2}\ln(U_{10})$,
$\kappa_{b}(\lambda =0.008)=5.956+0.072\cdot U_{10}+584.17/\sqrt{U_{10}}-495.24\cdot \exp(-U_{10})$.

The formulas work in the following interval of wind speeds *U*_10_ ∈ [3 m/s, 20 m/s].

**Figure 3.**
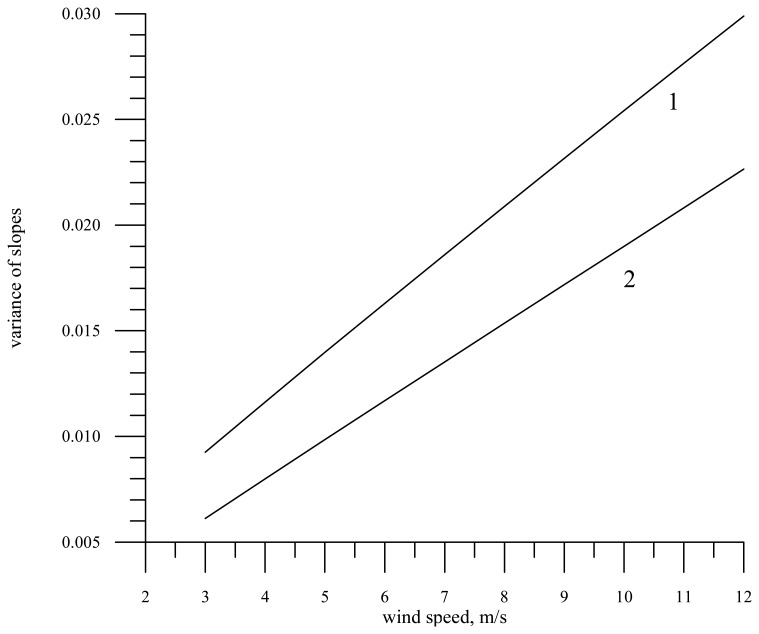
Dependence of the variance of large-scale wave slopes (the electromagnetic wavelength is 0.008 m) on the wind speed. Curve 1 – calculations for the spectrum on clean water, curve 2 – for the slick at the film elasticity *E* = 20 mN/m.

The slope variance of large-scale waves $\sigma_{xx}^{2}$ versus the wind speed for the wavelength 0.008 m in the absence of a slick (curve 1) and in the slick for the film elasticity *E* = 20 mN/m (curve 2) is plotted in fig. 3.

As is expected, the calculations exhibit that the slick influence on the statistical characteristics of waves is much stronger for shorter radio waves (0.008 m), than for longer ones. For 0.008 m the slope variance in the slick is reduced by about 30%, while for 0.055 m by less than 4%.

Other statistical characteristics of ripples in the slick are less variable. The variance of orbital velocities is mainly determined by large-scale waves, thus, e.g., for the wind speed 7 m/s and the wavelength 0.008 m the difference in the variance of orbital velocities is less than 1%.

The variance of sea wave heights is fully determined by the large-scale component of the wave spectrum and does not change in the slick.

## 4. The Doppler spectrum of a backscattered microwave signal

In contrast to the radar backscattering cross section, the Doppler spectrum of a reflected radar signal is not used now to detect sea surface slicks.

As is known, the Doppler spectrum width of a fixed radar at small incidence angles (∼ 0° – 15°) primarily depends on the variance of orbital velocities. This results in lower sensitivity of the Doppler spectrum to the slick occurrence on water surface in comparison with the radar backscattering cross section.

In the range of middle incidence angles (∼20° -60°) the Doppler spectrum is also low sensitive to the presence of slicks on the surface, because large-scale waves only modulate the reflected signal, while the Doppler spectrum width depends on the variance of orbital velocities. Therefore, disinterest to the use of the Doppler spectrum for slick detection seems to be quite feasible at first sight.

In [29, 30] we have shown that the Doppler spectrum can be sensitive to the slick occurrence when measurements are carried out in the transition range of incidence angles (16° −21°). In this case both the Bragg and quasi-specular scattering mechanisms participate in the reflected signal formation.

**Figure 4.**
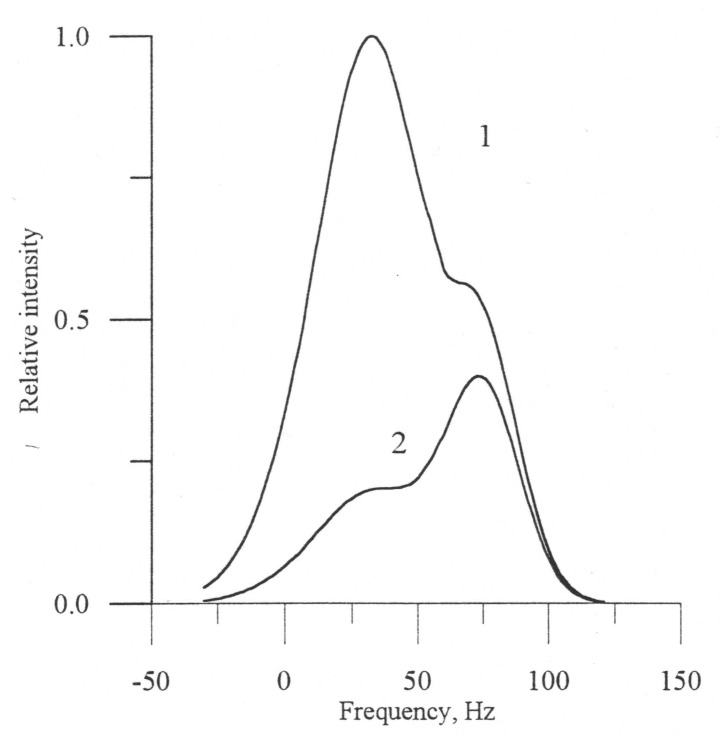
The X-band radar Doppler spectra for clean surface (1) and a slick (2) at wind speed 6 m/s (JONSWAP sea spectrum model) at 22° incidence angle [29].

If in the absence of a slick the Bragg scattering mechanism predominates, the total spectrum only slightly shifts with respect to the carrier frequency (curve 1, fig. 4).

Resonance ripples are suppressed in the slick and the power of the Bragg component considerably decreases, which provides domination of the quasi-specular component in the total Doppler spectrum. The quasi-specular component shifts more strongly in relation to the carrier frequency in comparison with the resonance scattering, due to the difference of the scattering mechanisms. The shift of the Doppler spectrum depends on the phase velocity of large-scale waves at small incidence angles and on the phase velocity of resonant ripples at the middle incidence angles.

As a result, the total Doppler spectrum essentially varies in going from clean water to the film-covered part. This effect was confirmed experimentally [30]. Figure 4 (see page 8) shows a theoretical example of the Doppler spectrum variation in going from the slick (2) to clean water (1).

Further studies have shown that under some definite conditions the Doppler spectrum can be used to measure water surface slopes [9, 10], i.e., the most sensitive parameter to the SAS film occurrence on water surface. Consequently, the Doppler spectrum can “see” the slope variance in going from clean water to the film-covered part. Let us verify this assumption by numerical calculations.

**Figure 5.**
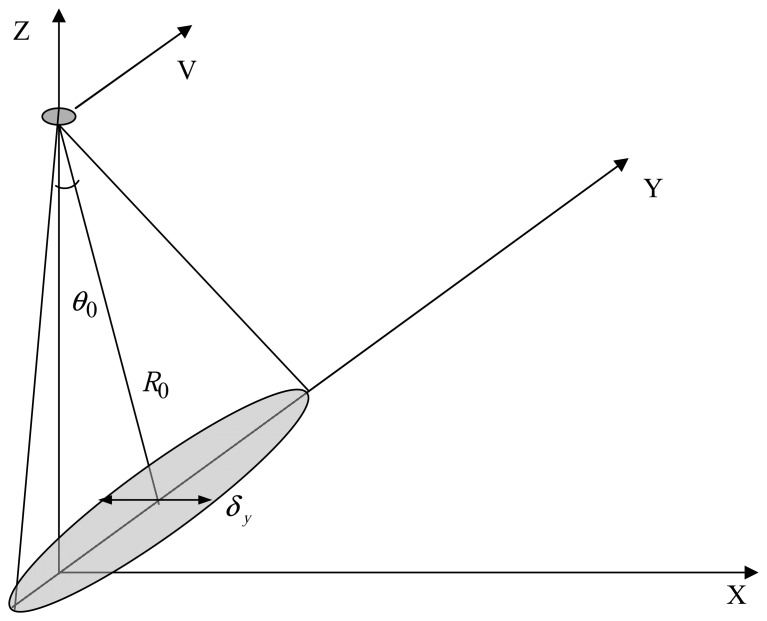
Measurement scheme.

Consider the experimental scheme presented in fig. 5. Radar moves with the velocity **V** in the ***YZ*** plane. As we examine small incidence angles, the quasi-specular scattering mechanism predominates. The antenna orientation is shown in the figure. The initial statement of the problem is discussed, e.g., in [31, 32].

Now we give the formula for the Doppler spectrum width at the level of -10 dB from the maximum in the considered measurement scheme [7, 8]:

(8) $$\Delta f_{10}=\frac{4\sqrt{\ln10}\cdot \cos\theta_{0}}{\lambda }\cdot {\left[\frac{1.38V^{2}}{R_{0}^{2}k^{2}\delta_{x}^{2}}+2\sigma_{tt}^{2}-\frac{2K_{xt}^{2}}{\sigma_{xx}^{2}}-\frac{2K_{yt}^{2}}{\sigma_{yy}^{2}}++\frac{2K_{xt}^{2}\delta_{y}^{2}}{\sigma_{xx}^{2}\left(5.52\sigma_{xx}^{2}\cos^{2}\theta_{0}+\delta_{y}^{2}\right)}+\frac{2\delta_{x}^{2}\sigma_{yy}^{2}}{\left(5.52\sigma_{yy}^{2}+\delta_{x}^{2}\right)}\cdot {\left(\frac{K_{yt}}{\sigma_{yy}^{2}}-V\right)}^{2}\right]}^{0.5},$$

where *λ* is the radar wavelength, *k* is the wavenumber, *R*_0_ is the distance to the center of the scattering area, and the flight height equals *H*_0_ = *R*_0_ cos*θ*_0_. The antenna beam pattern is assumed to be Gaussian, where *δ_x_* and *δ_y_* is the antenna beam pattern at the half-power level along the ***X*** and ***Y*** axes, respectively.

To describe a rough water surface, in addition to the slope variances ( $\sigma_{xx}^{2}$and $\sigma_{yy}^{2}$) and the orbital velocity variance( $\sigma_{tt}^{2}$) we use the following statistical characteristics: the coefficients of correlation between slopes and the vertical component of the orbital velocity *K_xt_* (τ) and *K_yt_* (τ) at the time τ=0. The slope correlation coefficient *K_xy_*(0) along the ***X*** and ***Y*** axes is zero if waves propagate along the axis ***X*** or ***Y***. The correlation coefficients are calculated by the formula:

$$K_{\alpha \beta }={0.5\frac{\partial^{2}K(\vec{\rho },\tau )}{\partial \alpha \partial \beta }|}_{\vec{\rho },\tau =0},$$

where *K*(*ρ⃗,τ*) is the correlation function of the sea wave heights.

The Doppler spectrum shift *f_sh_* at a quarter turn of the antenna (the incidence angle is in the plane ***YZ***) is yielded by the formula:

$$f_{sh}=\frac{\sin\theta_{0}}{\lambda }\left(2V-\frac{2K_{yt}}{\sigma_{yy}^{2}}+\left(\frac{K_{yt}}{\sigma_{yy}^{2}}-V\right)\frac{2\delta_{x}^{2}}{\left(5.52\sigma_{yy}^{2}+\delta_{x}^{2}\right)}\right)$$

At nadir probing the shift is zero, while at oblique probing in measurements of a moving carrier the Doppler spectrum is very sensitive to the incidence angle. As a result, even a small inaccuracy in determining the incidence angle causes a large error in retrieval of the scattering surface parameters using the algorithms based on the Doppler spectrum shift. The algorithms using the Doppler spectrum width are more stable.

Now we consider the contribution made by the orbital velocity variance to the Doppler spectrum width for radars with knife-beam and narrow-beam antenna patterns.

**Figure 6.**
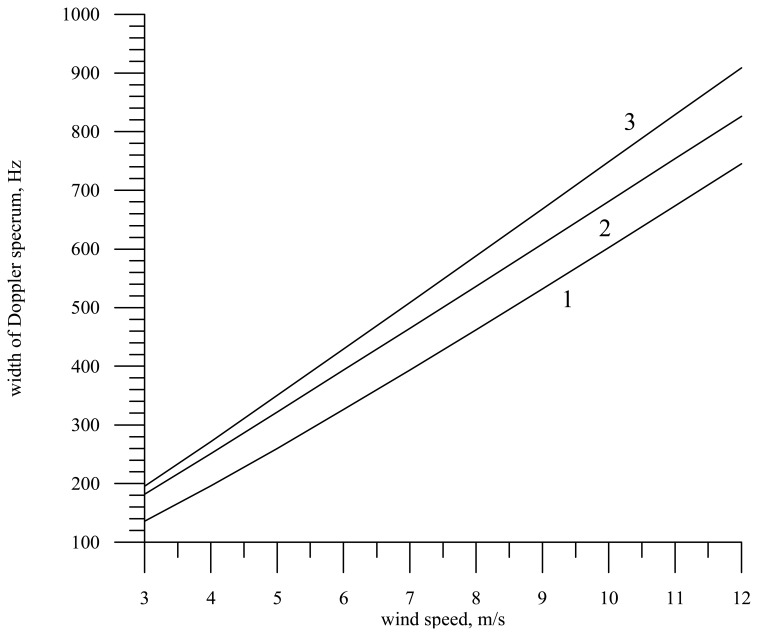
Dependence of the Doppler spectrum width on the wind speed for an immobile radar. The first line shows the case of a narrow-beam antenna, the second line shows the case of a knife-beam antenna, and the third line is the case *K_xt_* = *K_yt_* = 0.

Figure 6 plots the Doppler spectrum width versus the wind speed for radars with a narrow-beam antenna pattern (line 1) and with a knife-beam pattern (line 2). It is assumed in the calculations that the radar wavelength is 0.008 m and the incidence angle is zero. Line 3 shows the dependence in the absence of correlation between the vertical component of the orbital velocity and surface slopes (*K_xt_* (*τ*)= *K_yt_* (*τ*)= 0); note that it is the same for radars with knife-beam and narrow-beam antenna patterns.

Within the limits of the Kirchhoff method, backscattering occurs in the wave profile parts oriented perpendicularly to incident radiation, thus due to the correlation between slopes and orbital velocities not all orbital velocities contribute to the Doppler spectrum of a reflected radar signal but only the velocities “coupled” (by the incidence angle) with the reflecting part of the wave profile. The consequence of this fact is the limitation of the Doppler spectrum width.

An increase of the antenna beam width extends the range of slope angles of the wave profile, which contribute to the reflected signal and hence broaden the Doppler spectrum. That is why the Doppler spectrum width for radar with a narrow-beam antenna is smaller than that for radar with a knife-beam antenna.

The limiting case is the absence of correlation between slopes and orbital velocities. This was determined by the calculations with the zero correlation coefficient. In the figure this case is shown by line 3. The Doppler spectrum width becomes maximal and principally does not depend on the antenna beam width, i.e., the Doppler spectrum widths for radars with knife-beam and narrow-beam antenna patterns are the same.

For a moving radar the situation is different, because the Doppler spectrum width depends not only on the scattering surface self-motion but also on the carrier motion velocity. We assume in the numerical calculations that the radar motion velocity is 30 m/s (helicopter) and *λ* = 0.008 m.

**Figure 7.**
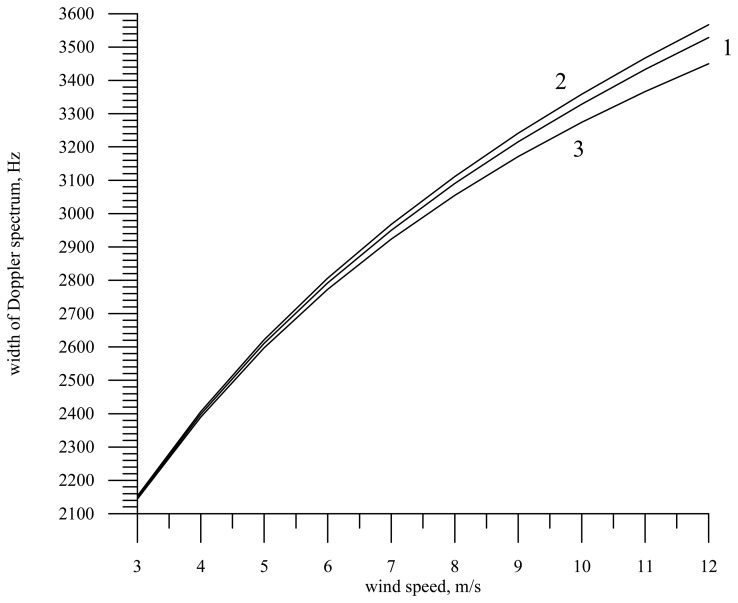
Dependence of the Doppler spectrum width on the wind speed for radar with a knife-beam antenna (22° × 1°). Curve 1 – developed wind waves, curve 2 – the absence of correlation between slopes and orbital velocities, and curve 3 – calculation results for an immobile surface, i.e., for the case $K_{xt}=K_{yt}\sigma_{tt}^{2}=0$ and a moving radar.

The dependence of the Doppler spectrum width on the wind speed under the assumption that the antenna pattern is knife-beam and waves propagate along the Y axis is shown in fig. 7.

As the wind speed increases, the Doppler spectrum width grows (curve 1). If it is assumed that the correlation coefficient of slopes and orbital velocities is zero, the Doppler spectrum width slightly increases since radar “sees” all orbital velocities (curve 2).

From the physical point of view this is explained by the fact that the wind speed growth leads to an increase of the surface slope variance and hence the reflected signal arrives at larger incidence angles, has a larger Doppler shift (projection of the radar motion velocity on the probing direction), but remains in the limits of the antenna directivity pattern.

If we deal with an immobile surface repeating the sea surface shape, then we obtain a similar dependence for a wide-beam antenna pattern (curve 3). As is seen in the figure, the Doppler spectrum width slightly decreases because self-motion of the surface is absent. In this case the Doppler spectrum width is fully determined by the surface slope variance.

Therefore, making measurements above land e.g., above desert, one can retrieve the ground slope variance by means of radar with a knife-beam antenna pattern.

If we deal with an immobile surface, the situation with a narrow-beam antenna radically changes. For the beam width of one degree, reflectors are the surface parts oriented perpendicularly to the incident radiation, i.e., practically horizontally. These parts are always present on the surface and the wind speed variance does not affect their existence. The radar backscattering cross section changes, while the Doppler spectrum width remains practically constant. This result enables one to assume that radar with a narrow-beam antenna pattern will badly see slicks on the sea surface if measurements are made from a moving carrier.

This peculiarity of the reflected signal formation gives rise to the fact that at a low velocity of radar motion the main contribution to the Doppler spectrum width is made by the surface self-motion rather than by the projection of the carrier motion velocity. In particular, for radar with a narrow-beam antenna moving with the velocity 30 m/s at the wind speed 5 m/s, the Doppler spectrum width is equal to 353 Hz for the sea surface, 424 Hz in the absence of correlation between slopes and orbital velocities, and only 239 Hz for an immobile surface. The width of the Doppler spectrum for an immobile radar is equal to 260 Hz.

### 5. Analysis of the SAS film influence on the Doppler spectrum width

As is mentioned above, the task of the present paper is to determine the experimental scheme enabling one to detect SAS films on the sea surface.

Let us enumerate possible variants of the measuring equipment installation analyzed below. First of all, radar can be installed fixedly (sea platform, the height is about 30 m, ***V*** = 0 m/s), move with a slow velocity (helicopter, the height is about 100–200 m, ***V*** = 30 m/s) or move rapidly above the sea surface (aircraft, the height is 2000 m, ***V*** = 200 m/s). The variant of measurements from a satellite is not considered. Below we examine the cases of oblique and nadir probing.

The Doppler radar antenna can be narrow-beam (*δ_x_* = 1° and *δ_y_* = 1°) or knife-beam (*δ_x_* = 22° and *δ_y_* = 1°).

The correct choice of radar wavelength is of great importance, thus to single out promising radiation frequencies we consider three wavelengths *λ*: 0.008 m, 0.021 m, and 0.055 m. These wavelengths are rather frequently used in actual radar systems of remote sensing.

The scheme of the numerical experiment is as follows. First, measurements are carried out above the “clean” part of the surface and then above the slick. The detection task is to determine the presence of a film on the surface by the variation of the Doppler spectrum width.

**Figure 8.**
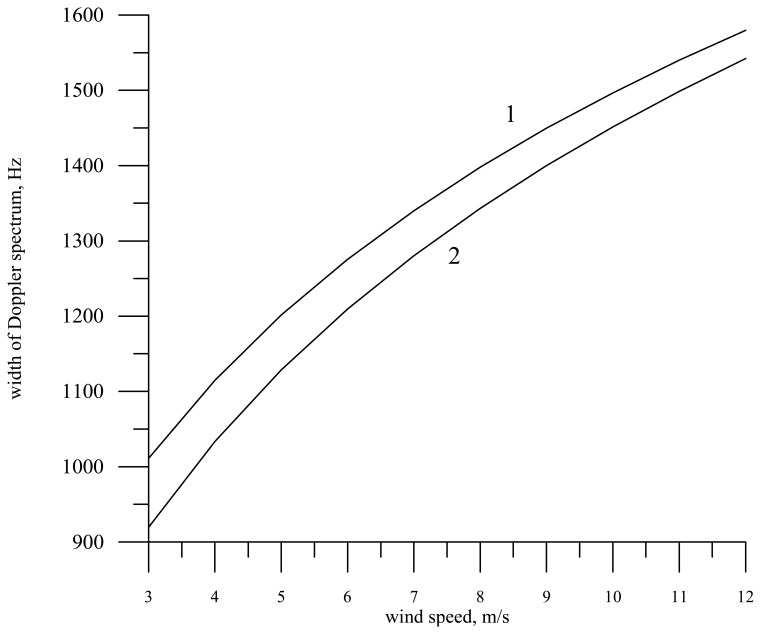
Dependence of the Doppler spectrum width at nadir viewing on the wind speed for the radar wavelength 0.021 m and the motion velocity 30 m/s. Curve 1 – without a slick, curve 2 – in the slick (a knife-beam antenna pattern).

As an example, in fig. 8 we present the dependence of the Doppler spectrum width at nadir viewing for a knife-beam antenna pattern (1° × 22°) and the wavelength 0.021 m on the wind speed in measurement from a helicopter for the clean water case (curve 1) and for the slick *E* = 20 mN/m (curve 2). In the calculations the direction of the helicopter motion (***V*** = 30 m/s) was chosen along the ***Y*** axis, while waves propagated counter to the ***Y*** axis, i.e., against the radar motion. Note that the Doppler spectrum width can be employed to unambiguously determine the direction of wave propagation [31].

The presence of the slick on the sea surface results in a decrease of the variance of large-scale wave slopes, and, consequently, in a decrease of the Doppler spectrum width in measurement from a moving carrier.

It is seen in the figure that the wind speed growth (roughness intensity) leads to the Doppler spectrum broadening. In this case the film influence is approximately equivalent to the decrease of the wind speed by 1 m/s.

Depending on the radar wavelength the division of the wave spectrum into large-scale waves and small ripples proceeds differently; the sensitivity of the Doppler spectrum width to the slick occurrence depends on the boundary wavenumber *κ_b_*. The larger the boundary wavenumber, the stronger the variation of the statistical characteristics of large-scale waves in the slick and hence the stronger the sensitivity of the Doppler spectrum.

For a detailed consideration we choose one wind speed (5 m/s) and make calculations for the three wavelengths. The calculation results for radar with a narrow-beam antenna pattern (1° × 1°) are presented in Table 1, the calculation results for radar with a knife-beam antenna pattern (1° × 22°) at nadir probing are presented in Table 2. Tables 3 and 4 deal with the calculations of the same parameters for oblique probing (the incidence angle is *θ*_0_ = 10°). The Doppler spectrum width Δ*f*_10_ in the tables is given in Hertz.

Note that for a narrow-beam antenna pattern (Table 1) at the radar wavelength of the order of 0.05 m and more, the Doppler spectrum width is not sensitive to slicks under arbitrary measurement conditions. For shorter waves the probability of slick detection is highest for an immobile radar and it diminishes as the motion velocity grows. In this case wind speed fluctuations can interfere.

The sensitivity of the Doppler spectrum width to a slick for radar with a knife-beam antenna pattern (Table 2) also considerably decreases at 0.055 m, hence shorter wavelengths should be used for the diagnostics.

Contrary to radar with a narrow-beam antenna pattern, the contrast of the “clean water”–“slick” transition increases in the Doppler spectrum width as the radar motion velocity grows.

This happens because in measurements by radar with a knife-beam antenna pattern mounted on a moving platform the main role in the formation of the Doppler spectrum width is played not by the orbital velocity variance but by the slope variance being most sensitive to the presence of SAS films on the surface.

At oblique incidence (Tables 3 and 4) the Doppler spectrum behavior in the range of quasi-specular scattering does not essentially vary, because the dependence of the Doppler spectrum width on the incidence angle is rather weak and proportional to cosine of the incidence angle. As is seen in Tables 3 and 4, the Doppler spectrum widths at the incidence angle 10° differ from those at nadir probing by a few per cent.

Thus radar with a knife-beam antenna pattern can be employed for diagnostics of slicks from a moving carrier.

Let us introduce a concept of contrast for the Doppler spectrum width in the following way:

(9) $$K_{ds}=\frac{\Delta f_{\text{slick}}}{\Delta f_{10}},\;$$

where Δ *f*_10_ Is the Doppler spectrum width on “clean water” and Δ *f_slick_* is the Doppler spectrum width in the slick.

**Figure 9.**
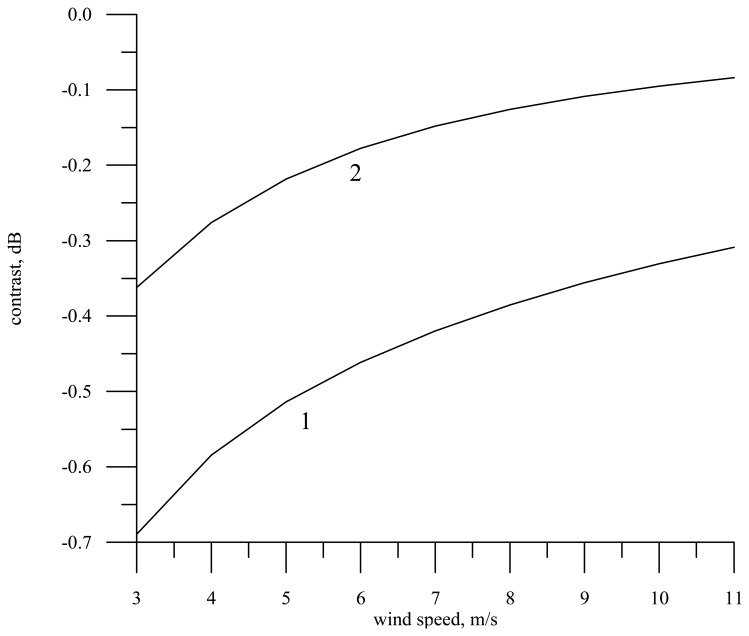
Dependence of contrast in the Doppler spectrum width on the wind speed for the radar wavelength 0.008 m and the motion velocity 200 m/s (nadir probing). Curve 1 – the contrast in the slick, curve 2 – the wind contrast caused by wind speed variation by 1 m/s (a knife-beam antenna pattern).

The dependence of the contrast on the wind speed for the radar wavelength 0.008 m and the motion velocity 200 m/s (a knife-beam antenna pattern) is shown by line 1 in fig. 9. It is assumed in the calculations that the film elasticity is *E* = 20 mN/m. To convert the contrast to decibel, the following formula is utilized: *K_dB_* = 10 · lg *K_ds_*.

As well as in conventional energy methods, a serious obstacle in diagnostics of surface films is wind speed variability. Near-surface wind is often unstable, which leads to fluctuations in the spectral density of small-scale waves and, as a result, to fluctuations in the reflected signal power and in the Doppler spectrum width.

To estimate this effect we calculated the wind contrast *K_wind_* caused by the difference between the wind speed at this point and the wind speed at the neighboring part by 1 m/s. The wind contrast was calculated as:

(10) $$K_{\text{wind}}=\frac{\Delta f_{10}(U_{10})}{\Delta f_{10}(U_{10}+1)}.\;$$

In fig. 9 the wind contrast is shown by line 2. For the assigned film parameters it is much weaker than the contrast in a slick.

**Figure 10.**
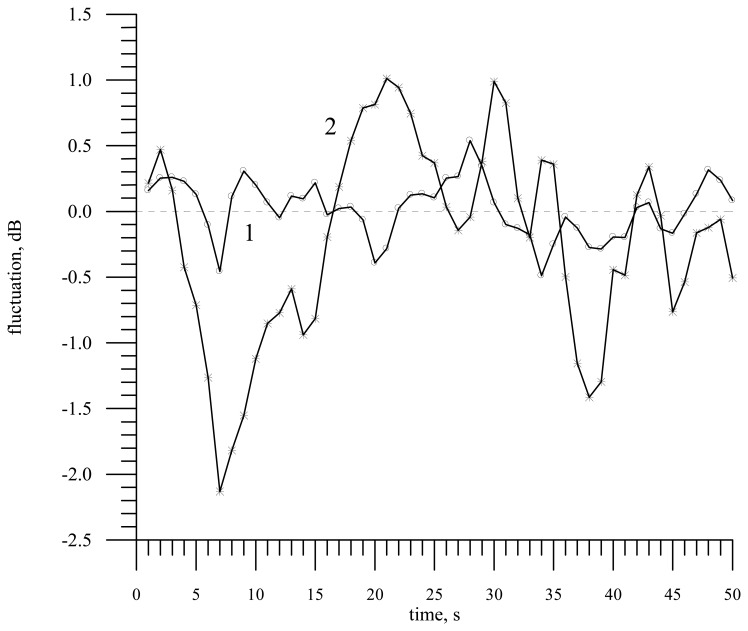
Fluctuations of the Doppler spectrum width (curve 1) and of the radar backscattering cross section (curve 2) during the flight above the Gorky water storage basin.

Though the absolute value of the contrast in the Doppler spectrum width is smaller than the absolute value of the contrast in the radar backscattering cross section, one should take into account that fluctuations of the reflected signal power are much stronger. This is seen in fig. 10 displaying as an example of variability the measurement results obtained during the flight above the Gorky water storage basin [31]. The observed contrasts in the power and in the Doppler spectrum width are attributed not to the slick presence but to variability of waves and wind speed above the sea surface in a small-size scattering area. For the flight height of about 150 m the size of the scattering area was 2.6 × 42.2 m at the half-power level. For a narrow-beam antenna the fluctuations could be even stronger.

Therefore, the question of advantages of the energy and spectral approaches to the slick detection remains open until field measurements are carried out.

### 6. Conclusion

The case of nadir probing is of interest to us, since radio altimeters have been used successfully in this range of incidence angles for a long time. The suggested design of radio altimeter with a knife-beam antenna pattern enables one to measure slopes of a rough water surface [33].

Besides the wind speed, the slope variance value can be also affected by the occurrence of SAS films on the sea surface; thus the developed systems can be used for slick diagnostics. The goal of the present paper is to validate this assumption.

The dependence of the Doppler spectrum width on the measurement conditions and a sea surface state is analyzed. In the numerical analysis we considered the influence of surface films on the Doppler spectrum width for radars with narrow-beam and knife-beam antenna patterns.

The calculations are based on the wave spectrum model taking into account the SAS film influence on the surface wave spectrum. The analysis has shown that among the statistical characteristics of large-scale waves the slope variance is most sensitive to the occurrence of SAS films.

In the case of a fixed radar it is possible to detect films at small incidence angles by using a narrow-beam antenna pattern. The calculations have demonstrated that radar with a knife-beam antenna pattern is poorly sensitive to the film presence. Note that the smaller the electromagnetic wavelength, the more sensitive the Doppler spectrum.

According to the calculations, radar with a narrow-beam antenna mounted on an aircraft (200 m/s) does not see the film, because the Doppler spectrum width is fully determined by the motion velocity of the carrier. This explains the point of view that the Doppler spectrum is inapplicable to slick diagnostics. However, our estimates have revealed that the employment of a knife-beam antenna changes the situation. In this case the Doppler spectrum width depends on the slope variance being rather sensitive to the SAS film occurrence on the surface

An additional advantage of a knife-beam antenna is that the reflected signal is collected from a larger area (from a larger number of scatterers), which decreases the signal fluctuations.

At an average motion velocity corresponding to that of our flights above the water storage basin (30 m/s), radar with a narrow-beam antenna is not practically sensitive to the film presence, while the sensitivity of radar with a knife-beam antenna remains satisfactory.

As the electromagnetic wavelength grows, the contrast of the “slick – clean water” transition weakens; hence the radar wavelength should not exceed 0.02 m.

The contrast in the Doppler spectrum width is weaker than the contrast in the radar backscattering cross section. However, the power fluctuations of the reflected signal are stronger than the fluctuations of the Doppler spectrum width, thus one cannot definitely state that the sensitivity of the Doppler spectrum in diagnostics (detection) of slicks on the sea surface is lower than the sensitivity of the radar backscattering cross section.

In a future experiment it is planned to make measurements above an artificial slick and to compare the sensitivities of the energy and spectral approaches to the slick detection.

## Annex 1

In oceanography, the frequency spectrum *S*_∑_ (*ω*) and the frequency-angular spectrum *S*_∑_ (*ω*)Φ*_ω_*(*ω*,*ϕ*) are usually measured, where *S*_∑_ (*ω*) describes the wave energy distribution over frequencies and Φ*_ω_*(*ω*,*ϕ*) is responsible for the angular distribution function.

A “radiophysical” model of the spectrum [19] exactly corresponding to the known experimental data was developed to describe wind waves.

In the frequency range from 0 to 1.2*ω_m_* the spectrum coincides with the JONWAP spectrum [20]:

$$S_{\sum }(\omega )=\alpha g^{2}\omega^{-5}\exp\left\{-1.25{\left(\frac{\omega_{m}}{\omega }\right)}^{4}\right\}.\gamma^{\exp\left[-{\left(\omega -\omega_{m}\right)}^{2}/\left(2\sigma_{*}^{2}\omega_{m}^{2}\right)\right]},$$

where *g* is the acceleration of gravity, $\kappa_{m}=0.697g/U_{10}^{2}$, and

$$\sigma_{*}\{\begin{array}{cc} 0.07, & \omega \leq \omega_{m};\\ 0.09, & \omega >\omega_{m}. \end{array}$$

The wavenumber *κ_m_* and the frequency *ω_m_* are related by the dispersion relationship for water waves.

At the frequency higher than 1.2*ω_m_* the wave spectrum is assigned by the formulas:

$\begin{array}{cc} S_{\sum }(\omega )=\frac{\alpha_{2}}{\omega^{4}}, & 1.2\omega_{m}<\omega \leq a_{m}\omega_{m} \end{array}$;
$\begin{array}{cc} S_{\sum }(\omega )=\frac{\alpha_{3}}{\omega^{5}}, & a_{m}\omega_{m}<\omega <\omega_{gc}≅64\;\text{rad}/\;\kappa =270\;\text{rad}\;/\text{m}) \end{array}$;
$\begin{array}{cc} S_{\sum }(\omega )=\frac{\alpha_{4}}{\omega^{2.7}}, & \omega_{gc}<\omega <\omega_{c}≅298\;\text{rad}/\text{s}(\kappa =1020\;\text{rad}\;/\text{m}) \end{array}$;
$\begin{array}{cc} S_{\sum }(\omega )=\frac{\alpha_{5}}{\omega^{5}}, & \omega_{c}<\omega \end{array}$.

The coefficients *α_i_* are calculated as follows:

$\begin{array}{ll} \alpha_{2}=S_{\sum }(1.2\omega_{m})\cdot {\left(1.2\omega_{m}\right)}^{4}, & \alpha_{3}=\alpha_{2}\cdot \alpha_{m}\omega_{m} \end{array}$,
$\begin{array}{ll} \alpha_{4}=\alpha_{3}\omega_{gc}^{-2.3}, & \alpha_{5}=\alpha_{4}\omega_{c}^{2.3} \end{array}$.

The value of the coefficient *a_m_* depends on the wind speed and is yielded by the expression:

a_m_ = 0.3713 + 0.29024 · U_10_ +0.2902/ U_10_.

To describe the angular distribution the following formula is used:

$\Phi_{\omega }=A\cdot \frac{2}{e^{2B\phi }+e^{-2B\phi }},-\pi \leq \phi \leq \pi$

where *B*=10*^b^* and

*b* = −0.28 + 0.65 · exp[−0.75 · ln(*κ/κ_m_*)]+0.01 · exp[−0.2+0.7 · lg(*κ/κ_m_*)].

The angle *ϕ*=*ϕ_T_* – *ϕ*_0_, where *ϕ*_0_ is the direction of wave propagation and *ϕ_T_* is the azimuth angle counted off from the ***X*** axis. The normalization coefficient *A* amounts to:

$$A=\frac{B}{\text{arctg}(\text{sh}2\pi B)}.$$

The developing waves are described by the concepts of the dimensionless wind fetch *x̃* and the dimensionless frequency *ω̃:*

$$\tilde{x}=xg/U_{10}^{2}\;\text{and}\;\tilde{\omega }=\omega \cdot U_{10}/g$$

where *x* is fetch in meters. When waves are developing from the shore under the action of wind, the fetch length coincides with the distance to the shore.

As waves develop, the following parameters of the spectrum model vary: *ω̃_m_, γ, α*. Below are the formulas for these values in the variation range of the dimensionless fetch *x̃* = [1430, 20170]:

${\tilde{\omega }}_{m}=0.61826+0.000003529\tilde{x}-0.00197508\sqrt{\tilde{x}}+62.554/\sqrt{\tilde{x}}-290.2/\tilde{x}$,
$\gamma =5.25366+0.000107622\tilde{x}-0.03776776\sqrt{\tilde{x}}-162.9835/\sqrt{\tilde{x}}+253251.5/{\tilde{x}}^{1.5}$,
$\alpha =0.0311937-0.002327736\ln(\tilde{x})-8367.9/{\tilde{x}}^{2}+4.51146\cdot 10^{617}\exp(-\tilde{x})$.

The fetch value *x̃* = 20170 corresponds to the fully developed wind roughness.

The present model of the roughness spectrum has been developed by analyzing experimental data for “radiophysical” application and is successfully used for solution of different problems.

## Figures and Tables

**Table 1.** The Doppler spectrum width for radar with a narrow-beam antenna (1° × 1°), Hz.

	*V* = 0 m/s		*V* = 30 m/s		*V* = 200 m/s	
Wave-length	*E*= 0 mN/m	*E* = 20mN/m	*E* = 0 mN/m	*E* = 20mN/m	*E* = 0 mN/m	*E* = 20mN/m
0.008 m	259.9	227.9	362.9	344.0	1626.6	1625.4
0.021 m	94.3	86.7	135.9	131.6	619.5	619.2
0.055 m	33.6	33.0	51.9	51.6	236.5	236.4

**Table 2.** The Doppler spectrum width for radar with a knife-beam antenna (22° × 1°), Hz.

	V = 0 m/s		V = 30 m/s		V = 200 m/s	
Wave-length	*E* = 0 mN/m	*E* = 20mN/m	*E* = 0 mN/m	*E* = 20mN/m	*E* = 0 mN/m	*E* = 20mN/m
0.008 m	321.9	320.0	3283.4	2964.5	20743.6	18428.5
0.021 m	122.3	121.9	1201.5	1128.7	7542.6	7015.6
0.055 m	46.6	46.5	436.2	429.8	2715.8	2670.3

**Table 3.** The Doppler spectrum width for radar with a narrow-beam antenna (1° × 1°), Hz at oblique probing (the incidence angle is 10°).

	V = 0 m/s		V = 30 m/s		V = 200 m/s	
Wave-length	*E* = 0 mN/m	*E* = 20 mN/m	*E* = 0 mN/m	*E* = 20 mN/m	*E* = 0 mN/m	*E* = 20mN/m
0.008 m	256.0	224.5	357.4	338.8	1601.9	1600.7
0,021 m	92.9	85.4	133.9	129.6	610.1	609.8
0,055 m	33.1	32.5	51.1	50.8	232.9	232.9

**Table 4.** The Doppler spectrum width for radar with a knife-beam antenna (22° × 1°), Hz at oblique probing (the incidence angle is 10°).

	V = 0 m/s		V = 30 m/s		V = 200 m/s	
Wave-length	*E* = 0 mN/m	*E* = 20 mN/m	*E* = 0 mN/m	*E* = 20 mN/m	*E* = 0 mN/m	*E* = 20mN/m
0.008 m	317.0	315.2	3233.5	2919.4	20428.4	18148.5
0.021 m	120.5	120.1	1183.2	1111.5	7428.0	6909.0
0,055 m	45.9	45.8	429.5	423.3	2674.6	2629.7
